# The life-cycle of the avian haemosporidian parasite *Haemoproteus majoris,* with emphasis on the exoerythrocytic and sporogonic development

**DOI:** 10.1186/s13071-019-3773-4

**Published:** 2019-11-04

**Authors:** Mikas Ilgūnas, Carolina Romeiro Fernandes Chagas, Dovilė Bukauskaitė, Rasa Bernotienė, Tatjana Iezhova, Gediminas Valkiūnas

**Affiliations:** Nature Research Centre, Akademijos 2, LT-08412 Vilnius, Lithuania

**Keywords:** *Haemoproteus*, Molecular characterization, Exoerythrocytic development, Megalomeronts, Sporogony

## Abstract

**Background:**

*Haemoproteus* parasites (Haemosporida, Haemoproteidae) are cosmopolitan in birds and recent molecular studies indicate enormous genetic diversity of these pathogens, which cause diseases in non-adapted avian hosts. However, life-cycles remain unknown for the majority of *Haemoproteus* species. Information on their exoerythrocytic development is particularly fragmental and controversial. This study aimed to gain new knowledge on life-cycle of the widespread blood parasite *Haemoproteus majoris*.

**Methods:**

*Turdus pilaris* and *Parus major* naturally infected with lineages hPHYBOR04 and hPARUS1 of *H. majoris*, respectively, were wild-caught and the parasites were identified using microscopic examination of gametocytes and PCR-based testing. Bayesian phylogeny was used to determine relationships between *H. majoris* lineages. Exoerythrocytic stages (megalomeronts) were reported using histological examination and laser microdissection was applied to isolate single megalomeronts for genetic analysis. *Culicoides impunctatus* biting midges were experimentally exposed in order to follow sporogonic development of the lineage hPHYBOR04.

**Results:**

Gametocytes of the lineage hPHYBOR04 are indistinguishable from those of the widespread lineage hPARUS1 of *H. majoris*, indicating that both of these lineages belong to the *H. majoris* group. Phylogenetic analysis supported this conclusion. Sporogony of the lineage hPHYBOR04 was completed in *C. impunctatus* biting midges. Morphologically similar megalomeronts were reported in internal organs of both avian hosts. These were big roundish bodies (up to 360 μm in diameter) surrounded by a thick capsule-like wall and containing irregularly shaped cytomeres, in which numerous merozoites developed. DNA sequences obtained from single isolated megalomeronts confirmed the identification of *H. majoris*.

**Conclusions:**

Phylogenetic analysis identified a group of closely related *H. majoris* lineages, two of which are characterized not only by morphologically identical blood stages, but also complete sporogonic development in *C. impunctatus* and development of morphologically similar megalomeronts. It is probable that other lineages belonging to the same group would bear the same characters and phylogenies based on partial *cytb* gene could be used to predict life-cycle features in avian haemoproteids including vector identity and patterns of exoerythrocytic merogony. This study reports morphologically unique megalomeronts in naturally infected birds and calls for research on exoerythrocytic development of haemoproteids to better understand pathologies caused in avian hosts.
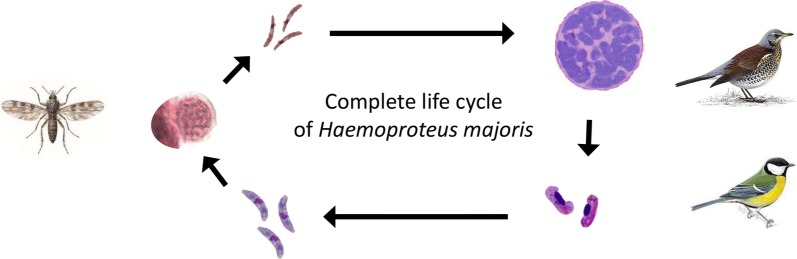

## Background

Blood parasites belonging to the genus *Haemoproteus* Kruse, 1890 (Haemosporida: Haemoproteidae) have been reported in birds all over the world, except for Antarctica. These are some of the most extensively studied pathogens of birds [1]. Two subgenera have been distinguished in this genus: *Haemoproteus* comprising species transmitted by louse flies (Hippoboscidae) and *Parahaemoproteus* comprising species transmitted by biting midges (Ceratopogonidae) [2]. Over 150 species belonging to this genus were described using morphological characters of their blood stages (gametocytes) [2–4] and available molecular data suggest that the number might be even greater [5–7].

Despite the cosmopolitan distribution and great species diversity, information about complete life-cycles of the vast majority of *Haemoproteus* parasites is lacking. This is particularly true for exoerythrocytic and sporogonic development of these pathogens [8, 9]. Numerous recent studies have addressed the taxonomy, genetic diversity, ecology, evolutionary biology and genetics of avian haemosporidians [1, 6, 7, 10–13]. Such studies were designed mainly by analysing the blood stages (gametocytes), which are present in the circulation and are relatively easy to sample. This provided opportunities to gain new knowledge on the molecular biology and ecology of these parasites, particularly their molecular diagnostics, however contributing scarce information about exoerythrocytic development in avian hosts and sporogonic development in vectors. Knowledge about these parts of the haemosporidian life-cycles is essential for better understanding the epidemiology of haemoproteosis and pathologies caused by these parasites but remains markedly fragmentary [14, 15]. Early studies usually considered *Haemoproteus* parasites as relatively benign in the vertebrate hosts [16]. However, the application of molecular diagnostic methods has challenged this opinion due to the discovery of numerous well-documented cases of severe haemoproteosis in non-adapted avian hosts [17–23]. Severe pathologies and even mortality have been reported, particularly when *Haemoproteus* infection was established in non-adapted (“wrong”) avian hosts. In such cases, exoerythrocytic development can be initiated but occurs incompletely and finally is aborted, yet it leads to severe disease [24]. Interestingly, sporogonic stages (ookinetes) of avian *Haemoproteus* parasites can markedly damage the midguts of blood-sucking insects (both vectors and non-vectors) and even kill them after blood meals with heavy gametocytaemia, but this issue and its biological significance remains insufficiently understood in wildlife [25].

Studies addressing the transmission and sporogonic development of avian haemoproteids remain uncommon [9, 14, 26–29] and vector species are unknown for the great majority of *Haemoproteus* species and their lineages [10, 30, 31]. Biting midges of the genus *Culicoides* have been successfully used in the experimental sporogony research of avian haemoproteids [9, 14]. These blood-sucking dipterans are abundant in temperate climate zones in Europe and wild-caught insects can be used for experimental exposure [14, 28]. Additionally, protocols have been developed to maintain *Culicoides nubeculosus* and some other species of biting midges in captivity, providing opportunities for experimental sporogony research [29, 32, 33]. Molecular detection of *Haemoproteus* lineages in wild-caught insects remains important because it provides useful information about links between parasite lineages and blood-sucking insects in the wild [34–37], but is insufficient to prove if the PCR-positive insects are competent vectors that can support complete sporogony and development of the infective sporozoites. Experimental observations and other methods providing opportunities to access sporozoites in insects remain essential in vector research [38].

This study aimed to contribute new knowledge about the genetic diversity, exoerythrocytic development and sporogony of *Haemoproteus majoris*, a widespread blood parasite of passeriform birds. One fieldfare *Turdus pilaris* naturally infected with the lineage hPHYBOR04 of *Haemoproteus majoris* was sampled and the parasites were identified to species level using the morphology of blood stages and partial cytochrome *b* (*cytb*) gene sequence. Exoerythrocytic development of this parasite is reported and sporogony followed in experimentally exposed *Culicoides impunctatus* biting midges. Because morphologically unique exoerythrocytic stages (megalomeronts) were detected, one great tit *Parus major* naturally infected with a closely related lineage hPARUS1 of *H. majoris* was also sampled and examined histologically for the presence of megalomeronts. The main goals of this study were: (i) to identify the gametocytes of the hPHYBOR04 lineage to the species level; (ii) to determine closely related lineages of this parasite; (iii) to investigate the exoerythrocytic development of the hPHYBOR04 lineage and to test a hypothesis that its closely related lineage hPARUS1 develop similar megalomeronts; (iv) to investigate the sporogonic development of the lineage hPHYBOR04 in experimentally exposed *C. impunctatus*.

## Methods

### Study site and selection of *Haemoproteus* species infected birds

Birds were sampled at the Ventės ragas ornithological station (55°20′38.93″N, 21°11′34.05″E) in May of 2018 and vector competence experiments were carried out in the Labanoras Forest (55°12′25.77″N, 25°55′26.47″E) in June of the same year in Lithuania. Birds were caught using mist nets, zig-zag traps and funnel type traps, which were available at the ornithological station. Blood was drawn from each individual bird by puncturing the brachial vein. Thin blood films were prepared on glass slides, fixed with absolute methanol, stained with Giemsa and examined under a microscope as described by Valkiūnas et al. [39]. In parallel, about 30 μl of blood from each individual bird was also collected and fixed in SET buffer [40] for polymerase chain reaction (PCR)-based testing. SET buffer-fixed samples were stored at − 4 °C in the field and at − 20 °C once back to the laboratory. One fieldfare *Turdus pilaris* and one great tit *Parus major* were found naturally infected with single *Haemoproteus* infections. These birds were selected for examination of blood stages, vector research and histological investigation. The status of single infection in experimental birds was determined by microscopic examination of blood films in the field and later confirmed by observations of electropherograms of DNA sequences in the laboratory (double-base calling was not reported).

### Design of sporogony research

Wild *Culicoides impunctatus* biting midges were exposed to *H. majoris* infection by allowing them to take blood meals on the selected fieldfare, as described and illustrated by Valkiūnas [2] and Žiegytė et al. [30]. Briefly, the infected fieldfare was held in hands protected by rubber gloves and biting midges were allowed to feed naturally between 22:00 and 23:00 h. This bird was exposed to bites at a site with high density of biting midges for approximately 30 min. The procedure was repeated 3 times in 3 successive days. *Culicoides impunctatus* willingly took bird blood meal on the parasite donor bird and numerous feeding insects were observed on the bird’s head. When approximately 20 females began taking blood meals on the bird’s head, the head with the feeding insects was carefully placed into an unzipped insect cage (approximately 12 × 12 × 12 cm) made of fine-mesh bolting silk. The engorged females flew off bird’s head after finishing the blood meal. The cage with engorged biting midges was then closed using a zipper. Males and non-fed females were removed from the cages. The cages with the engorged biting midges were transported to the laboratory where they were kept at 23 ± 1 °C, 70 ± 5% relative humidity and light:dark photoperiod of 17:7 h. The experimentally exposed biting midges were fed by placing one pad of cotton moistened with 10% saccharose solution on top of the cage. A total of 38 females were collected after blood meal on the fieldfare with intensity of mature gametocytes parasitaemia of 0.03%. The insects were dissected at set intervals of time for detection of ookinetes (5 insects), oocysts (6 insects) and sporozoites (27 insects), as described by Valkiūnas [2] and Žiegytė et al. [30]. Ninety-six non-fed females of *C. impunctatus* were collected at the study site and fixed in 96% ethanol. They were used to evaluate the possibility of natural infections in biting midges at the study site.

### Sporogonic stage samples

The experimentally exposed biting midges were dissected and preparations of ookinetes, oocysts and sporozoites were made at set time intervals. Briefly, the insects were anesthetized by placing them in a tube covered with a cotton-pad moistened with 96% ethanol. For visualizing the ookinetes, midguts of the blood-fed *C. impunctatus* females were extracted and gently crushed on objective glass slides 12 h post-exposure (hpe); thin preparations were made, fixed and stained the same way as blood films.

For oocyst observation, temporary preparations were made between 3–6 days post-exposure (dpe). Midguts were gently dissected and placed on a glass slide. Then, a drop of 2% mercurochrome solution was placed on each midgut, which was covered with a coverslip and examined under a microscope, as described by Žiegytė et al. [30].

To visualize sporozoites, the salivary glands were isolated from the biting midge females 6–9 dpe and gently crushed on glass slides to prepare small thin smears. The smears were fixed with absolute methanol and stained with 4% Giemsa solution for 1 h.

After each insect dissection, residual parts of their bodies were fixed in 96% ethanol and used for PCR-based analysis to confirm insect species identification and the presence of the corresponding parasite lineage in vectors. Dissection needles were disinfected in fire to prevent contamination after each dissection.

### Histological samples

At the end of the study, the naturally infected experimental fieldfare and one great tit infected with *H. majoris* (lineages hPHYBOR04 and hPARUS1, respectively) were euthanized. Brain, heart, kidneys, liver, lungs, spleen and a piece of the pectoral muscle were collected from each bird, fixed in 10% neutral formalin and embedded in paraffin blocks. Histological sections of 4 µm were prepared, stained with haematoxylin-eosin (H&E) and examined microscopically [2]. Additionally, histological sections of 4 µm were also prepared on paraffin membrane slides (MMI-MembraneSlide, Molecular Machines and Industries, Zurich, Switzerland) for laser microdissection studies.

### Parasite morphological analysis

#### Blood stages

An Olympus BX61 light microscope (Olympus, Tokyo, Japan) equipped with an Olympus DP70 digital camera and AnalySIS FIVE (Olympus Soft Imaging Solution GmbH, Münster, Germany) imaging software was used to examine blood slides, prepare illustrations and take measurements of gametocytes. The blood films were examined for 15–20 min at medium magnification (400×) and then at least 100 fields were studied at high magnification (1000×). Intensity of parasitaemia was calculated as a percentage by actual counting of the number of parasites per 1000 erythrocytes or per 10,000 erythrocytes if the infections were light [41].

#### Exoerythrocytic stages

An Olympus BX51 light microscope (Olympus) equipped with an Olympus DP12 digital camera and Olympus DP-SOFT imaging software was used to examine H&E stained histological sections. First, each histological preparation was examined at medium magnification (400×). If present, tissue stages of haemosporidian parasites can be readily visible. If exoerythrocytic meronts were found, they were examined and illustrated under set of different magnifications (100, 200, 400 and 1000×) for better visualization of parasite location and structure.

#### Sporogonic stages

An Olympus BX43 light microscope (Olympus, Tokyo, Japan) equipped with an Olympus SZX2-FOF digital camera and QCapture Pro 6.0, Image Pro Plus (Teledyne Imaging, Surrey, Canada) imaging software was used to examine ookinete, oocyst and sporozoite preparations. All preparations were examined under high (1000×) magnification. Images of parasites were collected and used for measurement using the program QCapture Pro 6.0 (Teledyne Imaging, Surrey, Canada).

### Molecular analysis

#### DNA extraction and PCR from blood samples

Total DNA was extracted from blood samples fixed in SET buffer using the standard ammonium-acetate protocol [42]. Partial mitochondrial cytochrome *b* (*cytb*) sequences were amplified using a nested-PCR protocol [40, 43]. The total volume of the PCR mix was 25 µl and it consisted of 12.5 µl of Dreamtaq Master Mix (Thermo Fisher Scientific, Vilnius, Lithuania), 8.5 µl of nuclease-free water, 1 µl of each primer and 2 µl of template DNA. The primer pair HaemNFI/HaemNR3 was applied for the initial PCR according to the protocol described by Hellgren et al. [40]. The primer pair HAEMF/HAEMR2 was applied for the second reaction according to the protocol by Bensch et al. [43]. Two microlitres of the first PCR product was used for the second PCR instead of genomic DNA. Nuclease-free water (negative control) and a *Haemoproteus* sample, which was positive in previous testing (positive control), were used to determine possible false amplifications. No case of false amplification was found.

#### Laser microdissection, DNA extraction and PCR using single megalomeronts

**Fig. 1:**
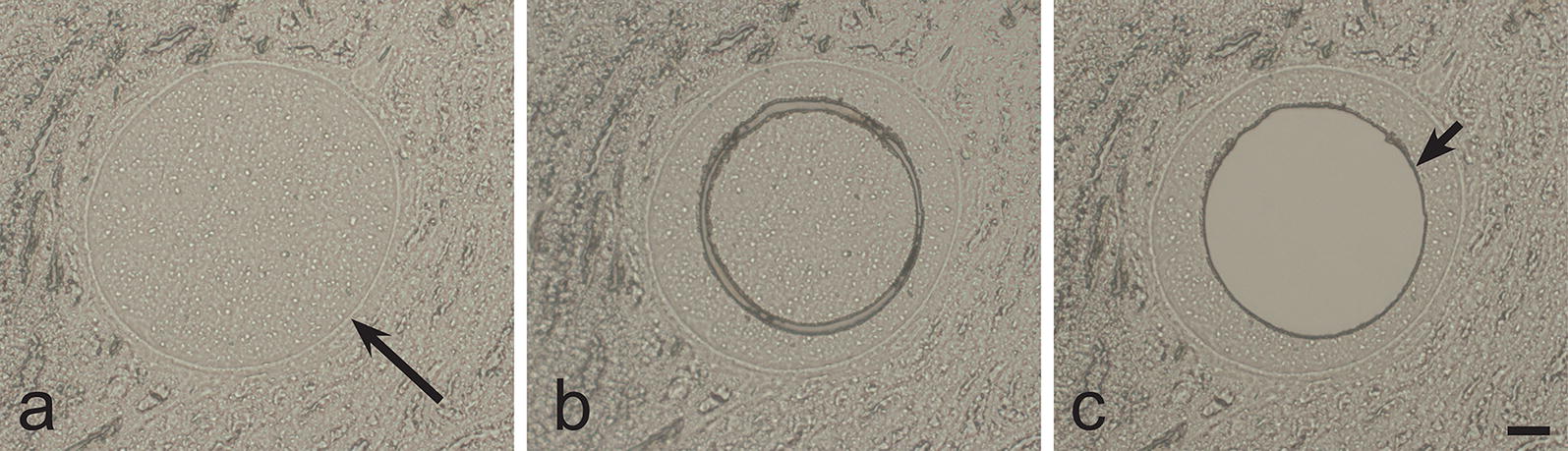
Laser microdissection of single megalomeronts of *Haemoproteus majoris* (lineage hPARUS1) from kidneys of the great tit *Parus major*. **a** Single intact megalomeront on a membrane slide before dissection. **b** Same single intact megalomeront on a membrane slide after dissection. **c** Same single megalomeront on a membrane slide after the removal of its dissected part. Note that only the central portion of the megalomeront was dissected for genetic analysis as was evident due to the intact capsule-like wall covering the parasite. Long simple arrow: capsule-like wall of megalomeront; short simple arrow: the hole in the membrane left after the dissection of the megalomeront. Unstained histological sections. *Scale-bar*: 20 μm

To confirm the identity of megalomeronts observed in the H&E stained histological section, laser microdissection was applied to isolate single megalomeronts. An Olympus IX71 light microscope (Olympus, Tokyo, Japan) equipped with Olympus/MMI CellCut Plus laser system and PTP function software (Predefined Target Position, Molecular Machines and Industries, Zurich, Switzerland) was used to cut single megalomeronts from non-stained histological sections. Adjustments of the contrast allowed for easy identification of these structures on paraffin membrane preparations (Fig. 1). Dissected megalomeronts were removed from the membrane using the adhesive silicone caps of the MMI IsolationCaps (Molecular Machines and Industries, Zurich, Switzerland) test tubes.

DNA from the dissected megalomeronts was isolated using the Chelex (Bio-Rad, Hercules, California, USA) DNA extraction protocol according to Palinauskas et al. [44]. Briefly, 0.2 g of Chelex was suspended in 1 ml of nuclease-free water and incubated in a 56 °C water bath for 1h. Next, the suspension was allowed to cool to room temperature. Then, 25 µl was placed on the wall of each tube, vortexing the suspension between each pipetting. After that, 0.7 µl of Proteinase K (10 mg/ml) was added to each Chelex drop. The tubes were then closed and flipped upside down for the Chelex/Proteinase K mixture to cover the adhesive cap of each tube. Tubes (still upside down) were incubated in a 56 °C water bath for 1h followed by 12 min incubation at 95 °C to inactivate the Proteinase K. The tubes were briefly spun down, the adhesive caps were replaced with regular caps and the tubes were centrifuged at 13.400× *g* for 12 min. The supernatant was used for PCR immediately. The total volume of the PCR mix was 25 µl and consisted of 12.5 µl of Dreamtaq Master Mix (Thermo Fisher Scientific), 8.5 µl of nuclease-free water, 1 µl of each primer and 2 µl of template DNA.

The primer pair HQF/HQR, which amplifies a short (194 bp) fragment of the mitochondrial *cytb* gene, was used for the PCR according to the conditions described by Ciloglu et al. [45]. The main goal of the amplification of the short sequences was to prove that the observed megalomeronts truly belong to *Haemoproteus* species. Due to the fixation of the histological samples in formalin and DNA extraction using the Chelex protocol, the relatively long haemosporidian parasite barcoding sequence (479 bp) of *cytb* could not be amplified. However, shorter fragments were amplified and used for the parasite genus identification. The primer pair HQF/HQR readily amplifies *Haemoproteus* spp. sequences [45] and was selected for the identification of megalomenronts to the generic level. Nuclease-free water (negative control) was used to determine possible false amplifications. No case of false amplification was found.

#### DNA extraction from vectors and PCR

DNA extraction from vectors and the applied PCR protocols were identical to those used for blood samples with one exception. Because vector samples for molecular analysis were stored in 96% ethanol, the fixed insects were transferred into SET buffer. This was achieved by placing the insect remains into an empty 1.5 ml tube, air-drying the remaining ethanol and pouring 250 µl SET buffer onto the dry insect.

Because the *C. impunctatus* used in the experiments were wild-caught, the prevalence of possible natural *Haemoproteus* spp. infection was determined in free-living biting midges that were sampled at the study site. These insects were tested by PCR-based methods. DNA was extracted from 24 pools of biting midges, each containing 4 flies. Additionally, remains of all experimental biting midges were collected after the dissections during sporogony research; they were fixed in 96% ethanol and tested by PCR amplification in order to confirm the identity of the parasite lineages in the experimentally exposed flies and confirm the species identification of biting midges. For the latter test, the insect specific primers LCO149 and HCO2198 were applied to amplify a fragment of the mitochondrial cytochrome *c* oxidase subunit 1 (*cox*1) gene [46].

### DNA sequencing and phylogenetic analysis

The success of the performed PCRs was evaluated by running electrophoresis on a 2% agarose gel. Two microlitres of the PCR products were used to test the success of amplification for each performed reaction. Successfully amplified PCR products were sequenced from both 5’- and 3’-ends using dye terminator cycle sequencing (Big Dye). Sequencing was carried out using an ABI PRISM TM 3100 capillary sequencing robot (Applied Biosystems, Foster City, California, USA).

Sequences were edited and examined using the BioEdit software [47]. The ‘Basic Local Alignment Search Tool’ (megablast algorithm) was used to identify the amplified *cytb* sequences and *cox*1 sequences of insects in the NCBI GenBank [48] and the ‘Basic Local Alignment Search Tool’ of the MalAvi database was used to double-check the identification [5]. A Bayesian phylogenetic tree was constructed using partial mitochondrial *cytb* sequences (479 bp). A total of 41 *Haemoproteus* spp. and 11 *Plasmodium* spp. lineages were used to construct the tree. All known *H. majoris* lineages were used; these were obtained from the MalAvi database (http://130.235.244.92/Malavi/). Additionally, several lineages that are most similar to the known lineages of this parasite were selected using the National Center for Biotechnology Information (NCBI) BLAST algorithm. *Plasmodium* lineages were included in the analysis with the aim to show that these and *Haemoproteus* spp. sequences do not position randomly in the tree but form separate clades. One *Leucocytozoon* sp. lineage was used as the outgroup.

The phylogenetic tree was computed using the MrBayes version 3.1 software [49]. Best-fit model of evolution (GTR) was selected by the software MrModeltest 3.7 [50]. The analysis was run for a total of 10 million generations and sample frequency was set to every 100th generation. Before the construction of the consensus tree, 25% of the initial trees were discarded as ‛burn in’. The constructed phylogenetic tree was visualized in FigTree v1.4.3 [51]. Genetic distances between lineages were calculated using the Jukes-Cantor model of substitution, as implemented in the program MEGA 7.0 [52].

### Statistical analysis

Statistical analysis of mean lineal parameters of parasites was carried out using ‘R’ version 3.4.3 and packages *Rcmdr* and *RcmdrMisc* [53]

## Results

### Parasite lineages and phylogenetic analysis

**Fig. 2:**
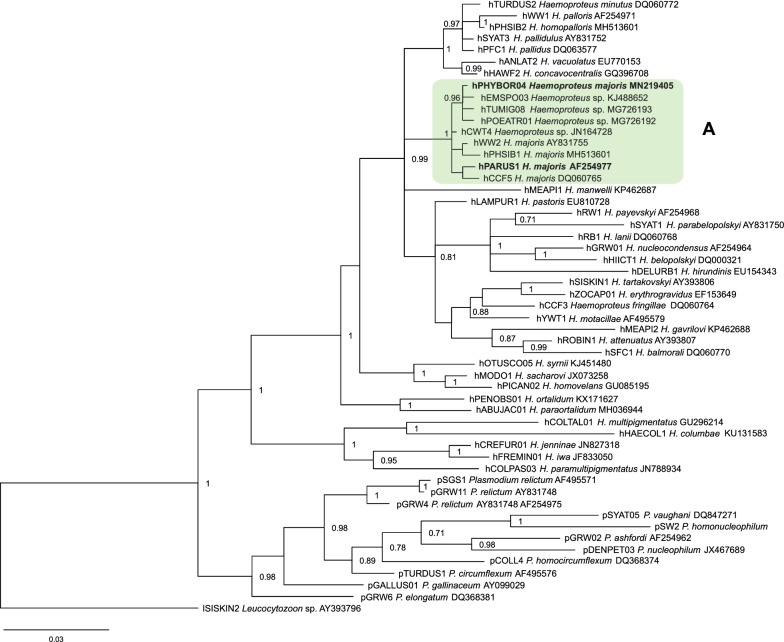
Bayesian phylogenetic tree constructed using 479 bp mitochondrial cytochrome *b* gene sequences of 41 *Haemoproteus* species and 11 *Plasmodium* spp. lineages, with one *Leucocytozoon* sp. lineage used as the outgroup. Posterior probabilities > 0.7 are indicated in the tree. MalAvi database codes of the lineages are given, followed by parasite species names and GenBank accession numbers. The green box indicates closely related lineages of parasites belonging to the *Haemoproteus majoris* group. Bold font indicates the lineages hPHYBOR04 and hPARUS1, which were investigated in this study

Single infection of the lineage hPARUS1 of *H. majoris* and the unidentified to the species level lineage hPHYBOR04 were found in the great tit and fieldfare, respectively, during PCR-based screening. This finding was in accordance with blood film microscopic examination, which allowed detecting the presence of single *Haemoproteus* infections of morphologically distinct gametocytes in these birds as well. Phylogenetic analysis grouped these parasite lineages in one well-supported clade (Fig. 2, Clade A) together with other lineages of *H. majoris* (hWW2, hPHSIB1 and hCCF5), suggesting a close phylogenetic relationship among them. Genetic differences among the morphologically identified *cytb* lineages of *H. majoris* varied between 0.2% (hPHYBOR04-hCWT4 and hCWT4-hWW2) and 1.3% (hPHYBOR04-hCCF5).

### Characterization of *Haemoproteus* (*Parahaemoproteus*) *majoris* (lineage hPHYBOR04)

To date, parasites of the lineage hPHYBOR04 have not been identified to the species level and their gametocytes have not been described. A description of the parasite is given below.

#### *Haemoproteus* (*Parahaemoproteus*) *majoris* (lineage hPHYBOR04)

- *Avian hosts*: According to this study, MalAvi database and the GenBank data, this lineage has been recorded in fieldfare *Turdus pilaris* (Lithuania; this study), Arctic warbler *Phylloscopus borealis* (USA, S. Galen & S. Perkins unpublished data, GenBank: MG726191) and Gray-cheeked thrush *Catharus minimus* (USA, S. Galen & S. Perkins unpublished data, MalAvi database).
- *Vector*: *Culicoides impunctatus* (see description of the sporogonic stages below).
- *Site of infection:* Gametocytes develop in mature erythrocytes; megalomeronts were seen in kidneys (see description below).
- *Representative blood films:* Voucher specimens (accessions 49045-49057 NS, intensity of parasitaemia 0.1–1.0%, *T. pilaris*, sampled at Ventes ragas, Lithuania, collected by M. Ilgūnas, from 17 May to 11 June 2018) were deposited at the Nature Research Centre, Vilnius, Lithuania and the Queensland Museum, Queensland, Australia (accession number G466217).
- *Representative histological sections*: Histological preparations of kidneys of *T. pilaris* are deposited at Nature Research Centre, Vilnius, Lithuania (accession number 49154 NS) and the Queensland Museum, Queensland, Australia (accession number G466218).
- *Representative vector preparations*: Preparations of ookinetes from midguts and sporozoites from salivary glands of *Culicoides impunctatus* are deposited at Nature Research Centre, Vilnius, Lithuania (accession numbers 49152 and 49153 NS, respectively).
- *Representative DNA sequences*: Mitochondrial *cytb* lineage hPHYBOR04 (479 bp, GenBank: MN219405).

### Description

#### Young gametocytes

**Fig. 3:**
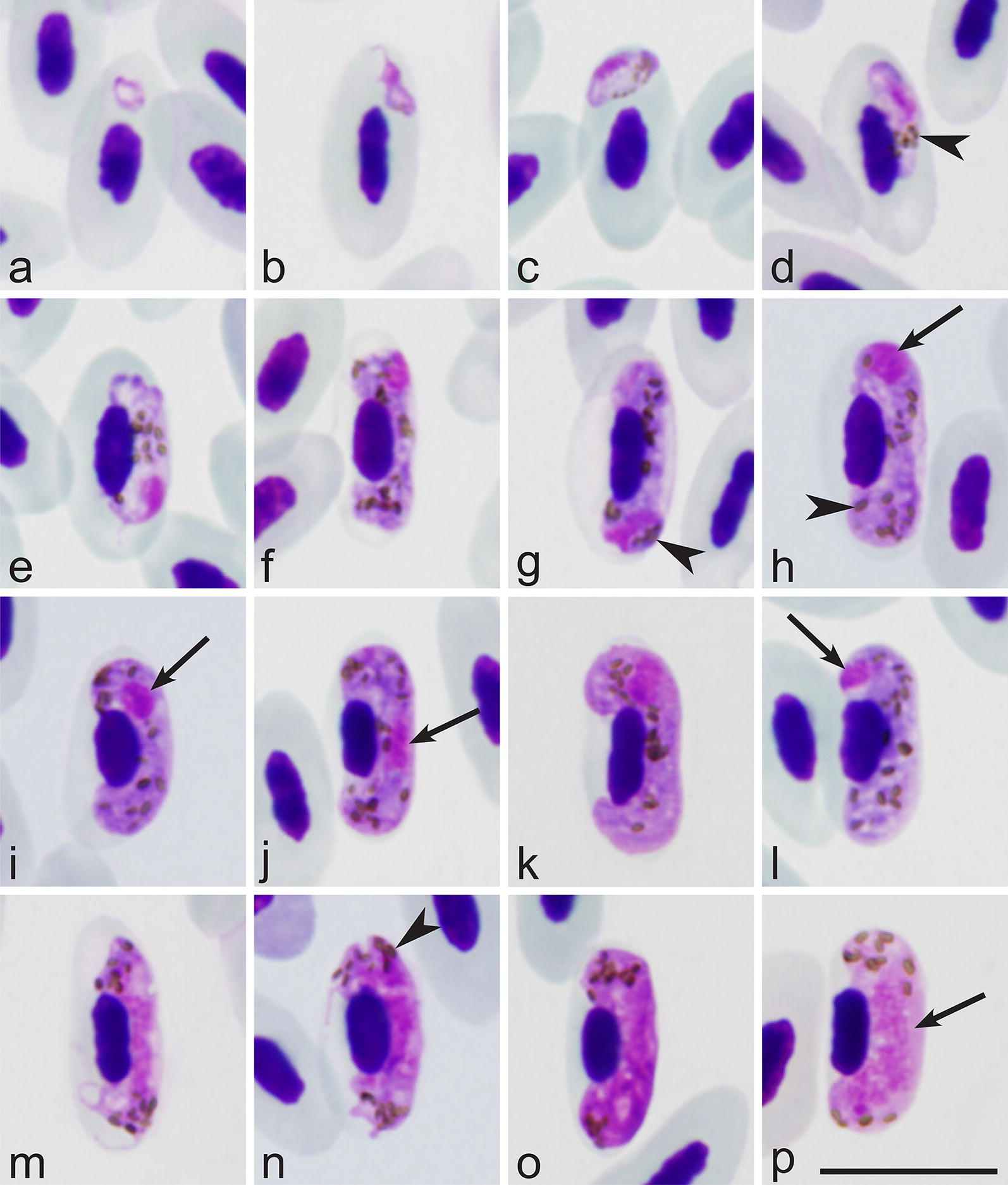
Gametocytes of *Haemoproteus majoris* (lineage hPHYBOR04) from the blood of the fieldfare *Turdus pilaris*. **a**–**d** Young gametocytes. **e**–**l** Macrogametocytes. **m**–**p** Microgametocytes. Arrows point to parasite nuclei; arrowheads point to pigment granules. Giemsa-stained thin blood films. *Scale-bar*: 10 μm

Earliest forms present free in cytoplasm, located anywhere in infected erythrocytes, but more frequently in polar or subpolar positions to erythrocyte nuclei (Fig. 3a-c). Gametocytes, reaching size of nuclei of erythrocytes in length, closely appressed to nuclei of infected erythrocytes (Fig. 3d) present, and this contact seen during gametocyte growth and maturation (Fig. 3e, f). Advanced gametocytes extended longitudinally along nuclei of erythrocytes and adhered to envelop of erythrocytes (Fig. 3e). Gametocyte nuclei prominent (Fig. 3c–e). Pigment granules small (< 0.5 mm), often grouped (Fig. 3e). Volutin granules visible and aggregated close to periphery in advanced gametocytes (Fig. 3e). Outlines varying from even (Fig. 3b, d) to irregular (Fig. 3a, e) and ameboid (Fig. 3b) present.

#### Macrogametocytes

Developing in mature erythrocytes. Cytoplasm blue, slightly heterogeneous in appearance, possessing fine homogenously dispersed volutin (Fig. 3l). Gametocytes growing along and closely adhering to nuclei of infected erythrocytes (Fig. 3f–h), slightly displacing nuclei laterally, enclosing them with ends, but not encircling completely (Fig. 3f–l) present. Advanced gametocytes closely appressed both to nuclei and envelope of erythrocytes and finally fill up poles of erythrocytes (Fig. 3h–l). Central part of growing gametocyte frequently constricted (Fig. 3f–h), giving dumbbell-like appearance to parasite (Fig. 3g, h); tips of dumbbell-shaped gametocytes adhering to envelope of erythrocytes (Fig. 3f–h). Dumbbell-shaped growing macrogametocytes common; after parasite maturation, dumbbell-shaped gametocytes not present anymore. Parasite nucleus variable in form, frequently roundish or oval (Fig. 3h, i), usually subterminal (Fig. 3h, i, k), but occasionally seen in central (Fig. 3j) or terminal (Fig. 3h, l) positions. Nucleolus not seen. Pigment granules of medium size (0.5–1 mm), roundish, oval and sometimes slightly elongate in form, usually more or less randomly scattered throughout cytoplasm (Fig. 3h–l). Outline of macrogametocytes usually even or slightly wavy, but slightly ameboid forms seen occasionally. Nuclei of infected erythrocytes slightly displaced laterally (Fig. 3h–l).

#### Microgametocytes

General configuration as for macrogametocytes with usual haemosporidian sexually dimorphic characters: pale stained cytoplasm and large, markedly diffuse centrally located nuclei. Ameboid growing microgametocytes (Fig. 3m) common, but fully-grown microgametocytes usually with even outline (Fig. 3o, p). Pigment granules aggregated in nucleus-free tips of gametocytes (Fig. 3p).

### Remarks

The main diagnostic characters of the reported gametocytes of lineage hPHYBOR04 in the fieldfare are indistinguishable from those of the lineage hPARUS1 belonging *H. majoris* in its type vertebrate host, the great tit. Parasites of both these lineages certainly belong to the same morphospecies.

### Exoerythrocytic development of *Haemoproteus majoris* (lineages hPHYBOR04 and hPARUS1)

**Fig. 4:**
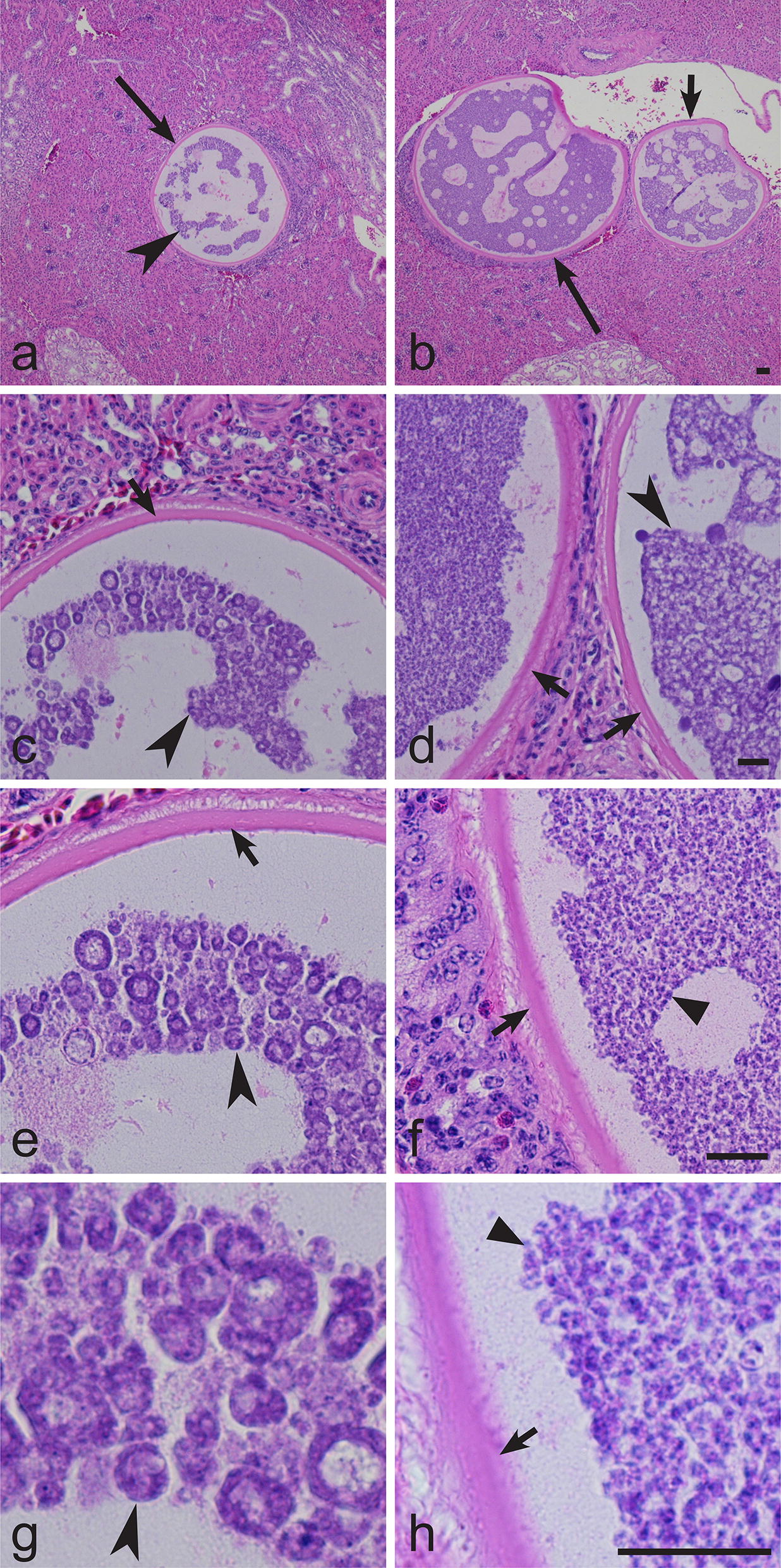
Megalomeronts of *Haemoproteus majoris* (lineage hPHYBOR04) from the kidneys of the fieldfare *Turdus pilaris* at four different magnifications. **a**, **b** 100×. **c**, **d** 200×. **e**, **f** 400×. **g**, **h** 1000×. Note that each megalomeronts is surrounded by a thick capsule-like wall and contains numerous cytomeres of irregular-shape, in which merozoites develop. Long simple arrows: megalomeronts; short simple arrows: capsule-like wall of megalomeront; simple arrowhead: cytomeres; triangular arrowheads: merozoites. Haematoxylin-eosin stained histological sections. *Scale-bar*: 20 μm

Exoerythrocytic meronts were seen only in the histological preparations of kidneys in fieldfare infected with *H. majoris* (lineage hPHYBOR04, 0.2% parasitaemia intensity) (Fig. 4). These were big (up to 360 μm in diameter) roundish bodies (Fig. 4a, b), each covered with a prominent capsule-like wall (Fig. 4c–f, h), which was up to 6 μm in width. Developing megalomeronts contained numerous irregularly shaped cytomeres (Fig. 4c, d), in which merozoites (Fig. 4f, h) develop. The smallest cytomeres were roundish, with nuclear material aggregated on their periphery (Fig. 4e, g). Mature megalomeronts contained numerous uninuclear merozoites (Fig. 4f, h). Host cell nucleus was not visible. Inflammatory reaction was not seen around the megalomeronts.

**Fig. 5:**
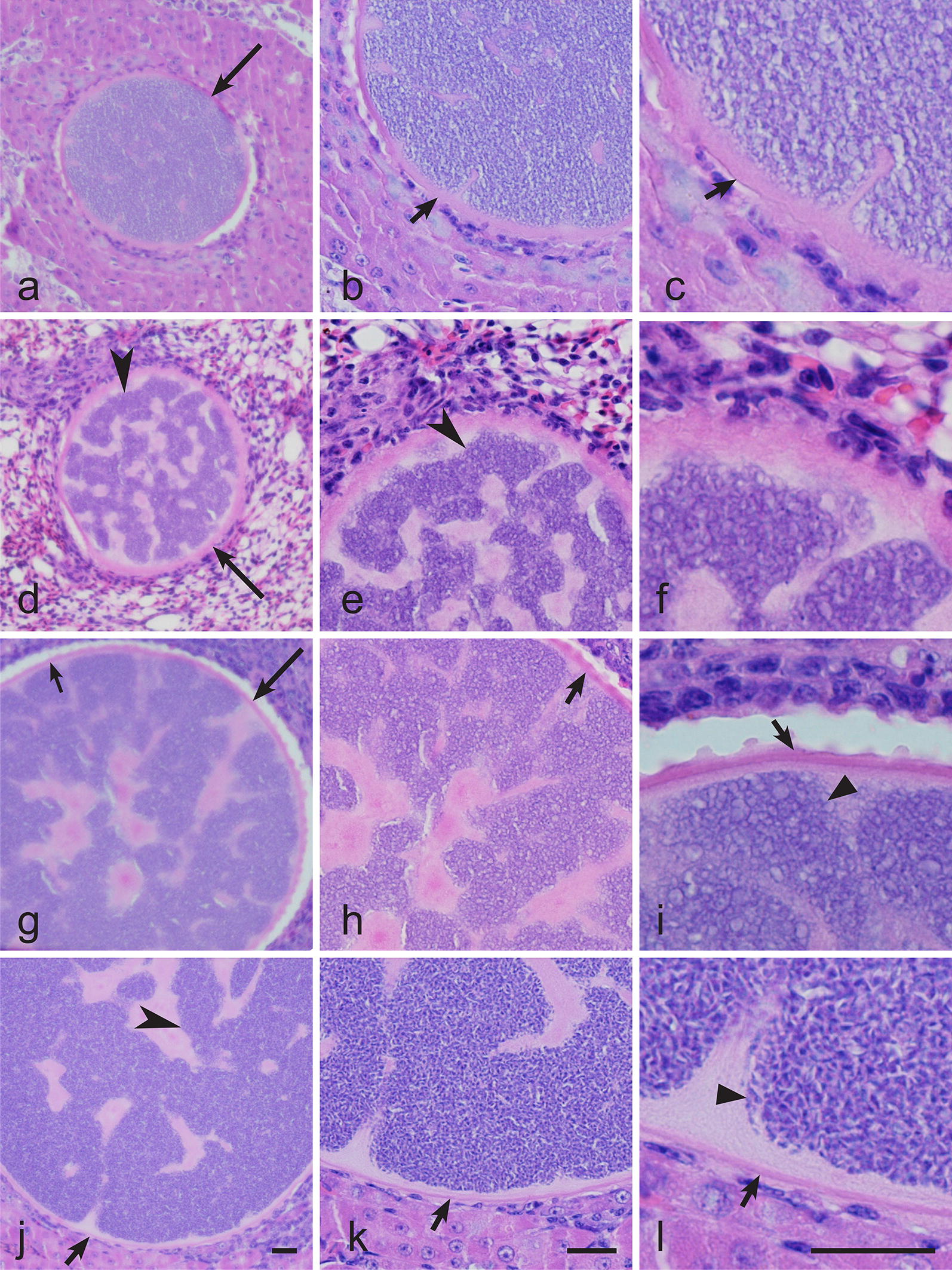
Megalomeronts of *Haemoproteus majoris* (lineage hPARUS1) from the internal organs of the great tit *Parus major*. **a**–**c** Liver. **d**–**f** Lungs. **g**–**i** Spleen. **j**–**l** Kidneys. Same megalomeronts are shown at three different magnifications: **a**, **d**, **g**, **j**, 200×; **b**, **e**, **h**, **k** 400×; **c**, **f**, **i**, **l** 1000×. Note that the structure of the megalomeronts was similar in different organs, i.e. the parasites were covered with prominent capsule-like walls and contained numerous irregularly-shaped cytomeres, in which merozoites develop. Long simple arrows: megalomeronts; short simple arrows: capsule-like wall; simple arrowhead: cytomeres; triangular arrowhead: merozoites. Haematoxylin-eosin stained histological sections. *Scale-bar*: 20 μm

Numerous morphologically similar megalomeronts were also observed in the internal organs of the great tit infected with *H. majoris* (lineage hPARUS1, intensity of parasitaemia of 4%) (Fig. 5). In this bird, single megalomeronts were seen in the liver, lungs, spleen; megalomeronts were especially numerous in the kidneys where up to 18 megalomeronts were observed in a single histological section (histological preparations of liver, lungs, spleen and kidneys of the *P. major* are deposited at Nature Research Centre, Vilnius, Lithuania (accession number 49155–49158 NS, respectively)).

Laser microdissection of single megalomeronts from the great tit (Fig. 1) followed by DNA extraction, PCR and sequencing, confirmed that the observed megalomeronts belong to *H. majoris*. Three individual megalomeront preparations were analysed using laser microdissection and the reported sequence (194 bp) coincided with the hPARUS1 lineage of genus *Haemoproteus* in all three cases.

### Sporogonic development of *Haemoproteus majoris* (lineage hPHYBOR04)

**Fig. 6:**
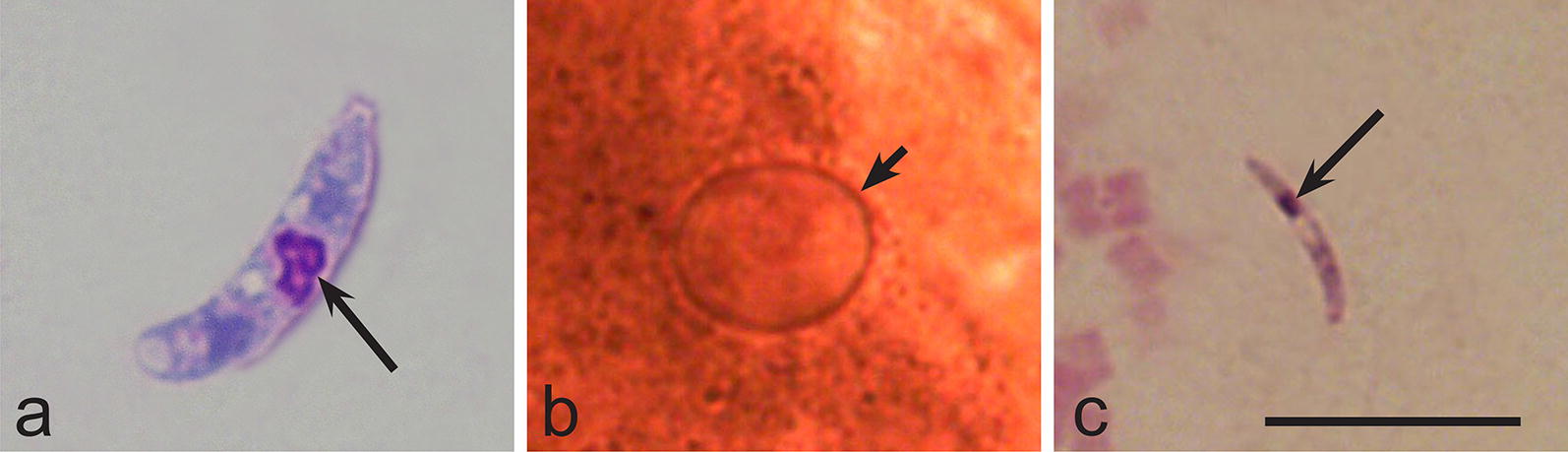
Sporogonic stages of *Haemoproteus majoris* (lineage hPHYBOR04) in the biting midge *Culicoides impunctatus*. **a** Mature ookinete. **b** Developing oocyst. **c** Mature sporozoite. Long simple arrows: parasite nuclei; short simple arrow: oocyst. **a**, **c** Giemsa-stained thin blood films. **b** Formalin-fixed whole midgut mount stained with hematoxylin. *Scale-bar*: 10 μm

All pools of wild-caught *C. impunctatus* females (controls) tested by PCR were negative for haemosporidian infections, indicating that the insects sampled in the wild population did not carry natural infections and thus could be used for experimental exposure and experimental sporogony research of *H. majoris*. *Haemoproteus majoris* (lineage hPHYBOR04) completed sporogony in the experimentally exposed biting midge *C. impunctatus*. Ookinetes, oocysts and sporozoites were observed (Fig. 6a–c). Ookinetes were detected 12 hpe (Fig. 6a). Mature ookinetes were elongated bodies with clearly visible prominent nuclei and vacuoles. The ookinetes (*n* = 6) measured 11.1–15.4 (mean 13.6 ± 1.3) µm in length and 1.9–2.8 (mean 2.3 ± 0.3) µm in width and 16.6–33.8 (mean 24.7 ± 5.7) µm^2^ in area. Developing oocysts were observed 4 dpe (Fig. 6b). Sporogonic development completed successfully and sporozoites were observed 6–9 dpe in the salivary gland preparations (Fig. 6c), indicating that these biting midges are likely the natural vector of this parasite. Sporozoites are fusiform bodies with slightly off-centre located nuclei. Measurements of the sporozoites are given in Table 1.

**Table 1:** Morphometry of sporozoites of *Haemoproteus majoris* (lineage hPHYBOR04) in biting midges *Culicoides impunctatus*

Feature	Measurements^a^
Length	6.7–9.2 (8.0 ± 0.6)
Width	0.9–1.6 (1.1 ± 0.2)
Area	4.4–8.5 (6.5 ± 1.0)

^a^Measurements (*n* = 21) are given in micrometres. Minimum and maximum values are provided, followed in parenthesis by the arithmetic mean and standard deviation

## Discussion

The key result of this study is the discovery of the exoerythrocytic stages in two lineages (hPHYBOR04 and hPARUS1) of *H. majoris*, a widespread blood parasite of passeriform birds. We have shown that these stages are megalomeronts (Figs. 4, 5). These data completed the information on the exoerythrocytic part of the life-cycle of this pathogen. To date, megalomeronts have been found in eight species of avian haemoproteids, but they were reported sporadically usually as case reports in non-passeriform birds [8]. Previous studies have detected megalomeronts in parrots [20, 24, 54], turkeys [55, 56], house sparrows [57–59], sacred kingfishers [60], bobwhite quails [17] and pigeons [61, 62] but parasite lineage identity has not been determined. As a result, the origin of the *Haemoproteus* species, which develop megalomeronts in avian hosts, remained unclear as well, except for *H. minutus* (lineages hTUPHI01 and hTURDUS2), which have been reported to cause lethal disease in parrots [24]. Particularly, it remained unclear if megalomeronts develop only when sporozoites are injected in non-adapted (“wrong”) avian hosts, as was the case in parrots, or do these stages develop normally in the life-cycles of *Haemoproteus* species. The present study shows that megalomeronts are a normal part of exoerythrocytic development in common avian haemoproteids because the lineages hPHYBOR04 and hPARUS1 of *H. majoris* are widespread and their gametocytes have been often reported during natural infections (Fig. 3) [5], indicating a complete life-cycle in avian hosts. Due to the huge size of the megalomeronts and high intensity in some organs (kidneys), megalomeronts of *Haemoproteus* parasites are worthy of more attention for better understanding of the pathologies caused during haemoproteosis. Unfortunately, the information on history of the infections reported here (freshly acquired or relapsed infection) in examined birds is unknown because the birds were naturally infected and wild-caught, so that the timing of infection cannot be specified. Infected individuals were active and looked non-exhausted in captivity.

Exoerythrocytic stages of avian haemoproteids were reviewed by Valkiunas & Iezhova [8]. Morphological features of *H. majoris* megalomeronts are unique among the avian haemoproteids described to date, particularly due to the presence of a prominent thick capsule-like wall covering the parasites and the markedly developed irregularly shaped cytomeres (Figs. 4, 5). Due to these features, the parasites reported here were similar to megalomeronts of *Leucocytozoon* spp. [2] or *Besnoitia* spp. [16]. To prove that the parasite stages detected in our study truly belong to haemoproteids, laser microdissection of single megalomeronts was carried out and DNA was isolated, amplified and sequenced. Because the detected *cytb* partial sequence coincided with the corresponding segment of the lineage hPARUS1, it was clear that the megalomeronts are stages of this parasite. This method was useful in species identification of oocysts in malaria parasites [63] and can be recommended in megalomeront studies. Additionally, *in situ* hybridization using specific probes can be used for distinguishing *Haemoproteus* infections from other parasites inhabiting tissues of birds [15].

The origin of the host cells of megalomeronts remains unclear. Host cell nucleus was not visible inside or close to megalomeronts, as is the case in megalomeronts of other avian haemoproteids but is characteristic to *Leucocytozoon* species [8]. Because megalomeronts of the lineage hPHYBOR04 were detected in numerous different internal organs, it seems probable that they might develop in non-specialised cells such as reticuloendothelial cells; further studies are needed to answer this question.

Nine closely related *Haemoproteus* lineages with genetic divergence of 0.2–1.3%, were grouped in a well-supported clade (Fig. 2, Clade A); of these, four have previously been characterised by morphology of their gametocytes as belonging to *H. majoris* [64]. This study showed that the lineage hPHYBOR04 also belongs to this species group. It is important to note that two recent studies [65, 66] have attributed the hCWT4 lineage to *H. majoris.* This is in agreement with our phylogenetic analysis; however, morphological characterization of the hCWT4 lineage gametocytes is still needed for final proof of this conclusion because some morphologically distinct *Haemoproteus* species differ just in few nucleotides in their partial *cytb* gene lineages [4, 29, 67].

Two lineages of *H. majoris* (hPHYBOR04 and hPARUS1) completed sporogonic development in *C. impunctatus* ([14]; this study). Previous studies have shown that *C. impunctatus* supports the complete sporogonic development of *H. balmorali* (lineage hSFC9), *H. belopolskyi* (hHIICT1), *H. minutus* (hTURDUS2), *H. motacillae* (hYWT1), *H. noctuae* (hCIRCUM01), *H. pallidus* (hPFC1) and unknown lineages of *H. dolniki*, *H. fringillae*, *H. lanii*, *H. parabelopolskyi* and *H. tartakovskyi* [2, 14, 30, 68, 69]. *Culicoides impunctatus* has been formerly considered to be mainly mammalophilic [70]; however, recent experimental observations and molecular testing indicate that this species also willingly feeds with bird blood and thus is worthy of attention in haemosporidiosis epidemiology research.

It is important to note that the lineages hPHYBOR04 and hPARUS1 of *H. majoris* are characterised by two similar features, i.e. these parasites complete sporogonic development in *C. impunctatus* and develop similar megalomeronts (Figs. 4, 5). This finding provides an opportunity to speculate that phylogenies based on the partial *cytb* gene can be used for prediction of life-cycle patterns in avian haemoproteids. In other words, it is probable that other lineages of Clade A (Fig. 2) would also complete sporogonic development in the biting midge *C. impunctatus* and develop megalomeronts in the vertebrate hosts. Testing of this hypothesis is of theoretical interest for better understanding of possible application of molecular phylogenies in studies of the biology of haemosporidian parasites.

## Conclusions

The complete life-cycle of *H. majoris* was uncovered, including the exoerythrocytic development, growth of gametocytes and sporogony from ookinetes to sporozoite stage. This study extended the knowledge about the genetic diversity of *H. majoris* by identification of one new lineage of this widespread blood parasite. We have shown that the lineage hPHYBOR04 of *H. majoris* completes sporogony in the biting midge *C. impunctatus*, as is the case with the lineage hPARUS1 of *H. majoris*. Importantly, these two lineages of *H. majoris* were found not only appearing in the same clade in the phylogeny, but also producing morphologically similar megalomeronts in different naturally infected avian hosts. In other words, the well-supported clades in phylogenies based on partial *cytb* gene are worthy of attention because they might indicate similar exoerythrocytic development in closely related parasites. This study shows that the megalomeronts of *Haemoproteus* parasites appear not only during abortive infection in non-adapted (“wrong”) avian hosts, but also develop during natural infections in the competent avian hosts. It is possible that megalomeronts often develop in various avian *Haemoproteus* infections thus, are worthy of more attention due to their large size, damage of internal organs and possible negative impact on the host health.

## Data Availability

Representative preparations of blood, exoerythrocytic and vector stages were deposited in the Nature Research Centre, Vilnius, Lithuania (accessions 49045-49057 NS and 49152-49158 NS). Additionally, preparations of blood and exoerythrocytic stages were deposited in the Queensland Museum, Queensland, Australia (accessions G466217 and G466218, respectively). A representative sequence was submitted to the GenBank database under the accession number MN219405. The datasets used and/or analysed during the present study are available from the corresponding author upon reasonable request.

## Abbreviations

*cytb*: mitochondrial cytochrome *b*
dpe: days post-exposure
H&E: haematoxylin-eosin
hpe: hours post-exposure
PCR: polymerase chain reaction
